# Large-Area, Flexible, Lead-Free Sn-Perovskite Solar Modules

**DOI:** 10.1021/acsenergylett.3c02066

**Published:** 2023-10-26

**Authors:** Wiktor Żuraw, Felipe Andres Vinocour Pacheco, Jesús Sánchez-Diaz, Łukasz Przypis, Mario Alejandro Mejia Escobar, Samy Almosni, Giovanni Vescio, Juan P. Martínez-Pastor, Blas Garrido, Robert Kudrawiec, Iván Mora-Seró, Senol Öz

**Affiliations:** † Department of Semiconductor Materials Engineering, Wroclaw University of Science and Technology, Wybrzeze Wyspianskiego 27, 50-370 Wroclaw, Poland; ‡ Saule Research Institute, Dunska 11, 54-427 Wroclaw, Poland; § Institute of Advanced Materials, Universitat Jaume I, Avenida de Vicent Sos Baynat, 12071 Castelló de la Plana, Spain; ∥ Saule Technologies, Dunska 11, 54-427 Wroclaw, Poland; ⊥ MIND-IN2UB, Department of Electronics and Biomedical Engineering, Universitat de Barcelona, Martí i Franquès 1, 08028 Barcelona, Spain; # UMDO, Instituto de Ciencia de los Materiales, Universidad de Valencia, Valencia 46980, Spain; ∇ Solaveni GmbH, Siemensstraße 42, 59199 Bönen, Germany

## Abstract

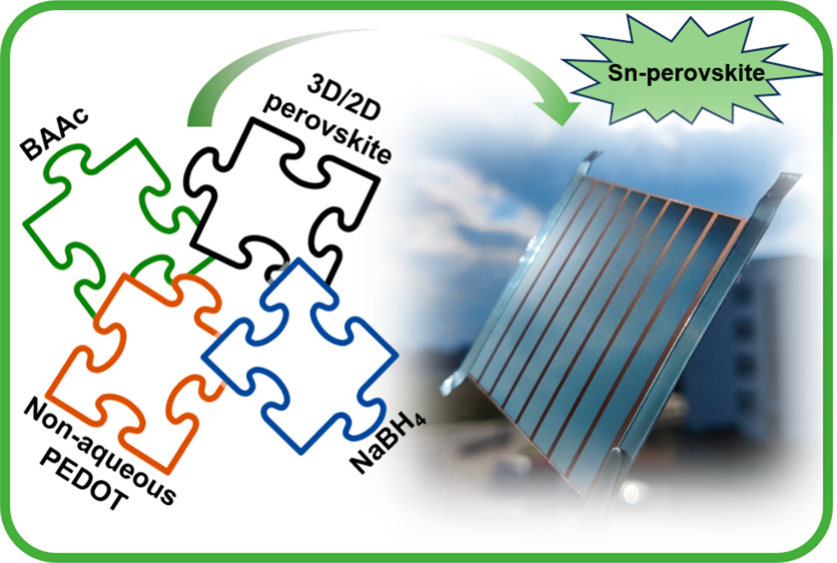

For the first time, large-area, flexible organic–inorganic tin perovskite solar modules are fabricated by means of an industry-compatible and scalable blade-coating technique. An 8-cell interconnected mini module with dimensions of 25 cm^2^ (active area = 8 × 1.5 cm^2^) reached 5.7% power conversion efficiency under 1000 W/m^2^ (AM 1.5G) and 9.4% under 2000 lx (white-LED).

Metal halide perovskites have gained attention over the past decade due to their promise in optoelectronic and photovoltaic applications. Perovskite solar cells (PSCs) have achieved up to 26% power conversion efficiency (PCE) for single-junction devices.^1^ Despite their excellent properties, Pb-based perovskites can be problematic in real-life applications due to concerns about their toxicity.^2^ Recently, much attention has been paid to Sn-based PSCs, with a reported PCE close to 15%.^3^ However, all the reports available in the literature refer to small-area cells made with non-scalable techniques such as spin-coating. Therefore, the development of methods that allow the fabrication of uniform, large-area thin films is a key step toward the commercialization of lead-free perovskite photovoltaics.

The main reason for the rather slow development of Sn-based perovskites is the easy oxidation of Sn^2+^ into Sn^4+^, which induces several degradation mechanisms and device performance losses.^4^ Another challenge is obtaining uniform layers, as the crystallization kinetics of Sn-based perovskites is faster compared to Pb analogues, producing non-uniform and pinhole-containing films.^5^ These facts could be addressed by using additives to reduce Sn^2+^ oxidation and to delay the crystallization, or by changing the solvent system, for example, increasing the dimethyl sulfoxide (DMSO) content.^6^ However, DMSO is known as a solvent that can accelerate Sn^2+^ oxidation. Even though alternative solvent systems have been proposed in the literature, the highest efficiencies have been obtained using DMSO mixed with dimethylformamide (DMF) or pure DMSO compositions.^7^ Moreover, these challenges are exacerbated when making large-area devices, as the antisolvent process cannot be used to promote crystallization, in contrast to small-area devices made by spin-coating.

Very recently we have successfully deposited FASnI_3_ Pb-free perovskite via a blade-coating technique for the first time. *N*-Butylammonium acetate (BAAc) was used as an additive to control the crystallization dynamics, allowing the fabrication of solar cells with an active area of 1 cm^2^ and a PCE of 3.7%.^8^ However, further scaling up requires module preparation with solar cells interconnected in series, and more material research is needed to improve the performance.

Here, we present the first report of blade-coated flexible, lead-free perovskite solar modules. We also illustrate how proper 3D/2D perovskite composition can help in the crystallization of the film, as well as how the choice of hole transport material (HTM) can drastically affect the final efficiency of the fabricated mini module. Finally, we demonstrate the potential of Sn-based perovskite solar devices with an achieved 5.7% PCE on a 25 cm^2^ flexible module for 1000 W/m^2^ irradiance (AM 1.5G) and 9.4% PCE for 2000 lx.

Experimental details are provided in the Supporting Information. Briefly, we aimed for a hybrid 3D/2D perovskite composition ((BA_0.5_PEA_0.5_)_2_FA_3_Sn_4_I_13_) with the addition of an ionic liquid (BAAc) and a reducing agent (NaBH_4_).^9−11^Figure 1a,b shows the structural and morphological characterization of the blade-coated perovskite layer. The XRD pattern shows the typical diffraction peaks for the (100) and (200) planes around 14° and 28° and additional signals below 5° representative of low-dimensional perovskite phases.^12^ Similarly, the photoluminescence spectrum displays not only the expected peak for 3D FASnI_3_ around 850 nm but also peaks at lower wavelengths that can be assigned to contributions from the quasi-2D phases, see Figure 1c.^12^ These emission bands are correlated with exciton resonances in the absorbance spectrum shown in Figure 1d.^12^

**Figure 1:**
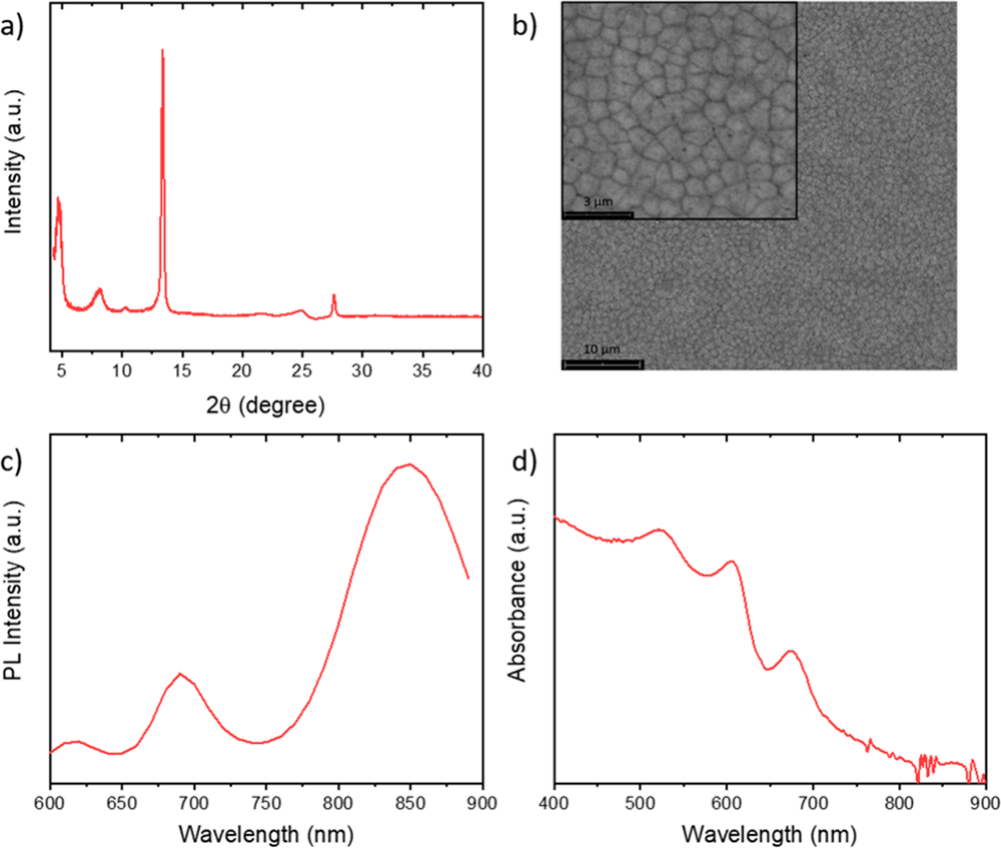
(a) X-ray diffraction pattern and (b) top-view scanning electron microscopy image of a blade-coated (BA_0.5_PEA_0.5_)_2_FA_3_Sn_4_I_13_ layer. (c) Normalized photoluminescence and (d) UV–vis absorbance spectra of the corresponding film.

Modules were fabricated in a p-i-n configuration with the following structure: polyethylene terephthalate (PET)/indium tin oxide (ITO)/HTM/(BA_0.5_PEA_0.5_)_2_FA_3_Sn_4_I_13_/C_60_/bathocuproine (BCP)/Ag. As HTM we used (1) an aqueous poly(3,4-ethylenedioxythiophene)–poly(styrenesulfonate) (PEDOT:PSS) dispersion, (2) a PEDOT dispersion in toluene, and (3) a nickel oxide dispersion (NiO_*x*_). For every HTM, we investigated the optimal P2 line processing conditions. P2 processing parameters are presented in Tables S1 and S2. Although PEDOT:PSS is the most widely used HTM in the fabrication of Sn-based PSCs, in our case, the maximum obtained efficiency was 1%. Despite using different laser powers during the P2 process, the short-circuit current density (*J*_sc_) obtained for each variant was very low, see Figure S1. We suspect that this is related to the re-absorption of water by the PEDOT:PSS during the P2 process, which was conducted in ambient conditions (25% RH).^13^ Current density–voltage (*J–V*) parameters for NiO_*x*_ are shown in Figure S2. We observed a significant increase in *J*_sc_ and fill factor (FF) compared to modules with PEDOT:PSS, but it is known that NiO_*x*_ can promote the oxidation of Sn-based perovskites and is also more prone to mechanical damage from bending, which is a significant parameter for the application of flexible solar cells.^14^

Recently, Di Girolamo et al. successfully employed a non-aqueous HTM source for Sn-based PSCs.^15^ With the PEDOT/Al_2_O_3_ bilayer we achieved a *J*_sc_ of 16.04 mA/cm^2^, and combined with an open-circuit voltage (*V*_oc_) of 4.85 V, this resulted in a PCE of 5.7% for the champion module under AM 1.5G illumination. A photograph of the fabricated module and forward scans for champion devices with varying HTMs are displayed in Figure 2a,b, and dark scans are plotted in Figure S3. The *J–V* parameters and *J–V* scans for the champion module are shown in Figures S4 and S5.

**Figure 2:**
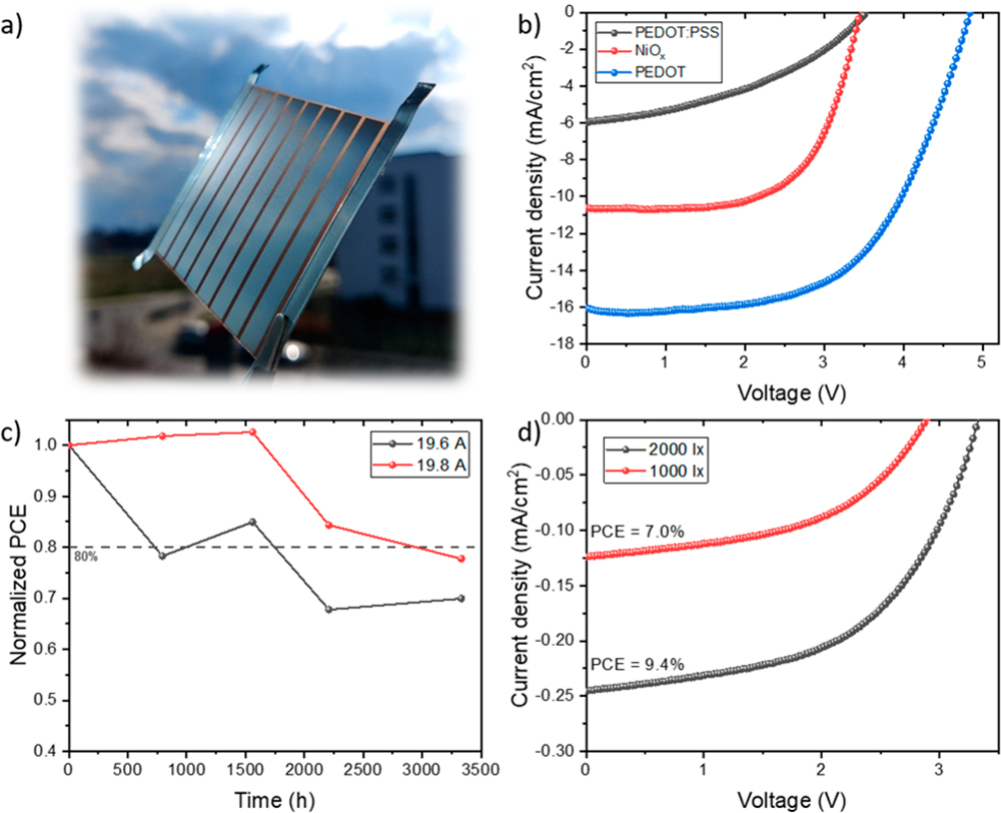
(a) Picture of the module, (b) *J–V* forward scans for different HTMs, (c) shelf-stability in N_2_ for the champion (19.6 A) and a module fabricated with higher P2 laser power (19.8 A), and (d) *J–V* forward scans for the champion module for 1000 and 2000 lx illuminance.

We also assessed the stability of our modules over time, see Figure 2c. Non-encapsulated samples were kept inside an N_2_-filled glovebox under dark conditions and were exposed to ambient conditions for *J–V* measurements periodically (30% RH). The laser power applied during the P2 patterning had a large impact on the module performance but did not play a significant role in the long-term durability. The difference in stability in the first 1600 h might relate to the air exposure time during P2 processing, but most modules achieved 80% of the initial efficiency (T_80_) after 2000 h, and after 3300 h they obtained similar values in the range of 70–80% of the initial PCE. The evolution of *J–V* parameters over time for the rest of the modules fabricated on PEDOT/Al_2_O_3_ is shown in Figure S6. A significant obstacle during the module fabrication is the degradation of the perovskite layer, which is exposed to the ambient atmosphere during the P2 process. Performing all the steps in an N_2_ atmosphere could improve the module’s final performance and stability even further, but this would be problematic for mass production, and a trade-off would be needed. Our results prove that, despite performing most of the steps in an ambient atmosphere—except for perovskite, C_60_/BCP, and Ag deposition—we were still able to obtain satisfactory module performance and stability, see Figure S7.

Finally, we tested the module behavior at low illuminance conditions, since recently there has been a growing interest in using PSCs for indoor applications.^16^ We achieved 7.0% and 9.4% PCE for 1000 and 2000 lx (366 and 738 μW/cm^2^), respectively, see Figure 2d. The measurement setup and absolute spectrum for each illuminance are shown in Figures S8 and S9. This result represents a significant milestone for indoor applications of flexible Sn-based perovskite modules and shows a path forward for further research on this topic.

In summary, we present the first report on Pb-free perovskite modules, formed by blade-coating on flexible substrates, demonstrating that Sn-based perovskite photovoltaic modules can be successfully prepared using scalable techniques. After optimization of the HTM/perovskite interface with a PEDOT/Al_2_O_3_ bilayer, the champion module achieved a power conversion efficiency of 5.7% under AM 1.5G 1 sun illumination and 9.4% under low light conditions.

## Supporting Information Available

Experimental methods, P2 process parameters, additional data, and characterization measurements (PDF)
