# Supplementary material for: Large-Area, Flexible, Lead-Free Sn-Perovskite Solar Modules
Source: ACS Energy Lett. 2023 Oct 26;8(11):4885–7. doi: 10.1021/acsenergylett.3c02066 (PMC10644357; doi:10.1021/acsenergylett.3c02066)
Supplement: Supplementary file 1 — nz3c02066_si_001.pdf [file nz3c02066_si_001.pdf]

Supporting information:

Large-area flexible lead-free Sn-perovskite solar modules

Wiktor Żuraw[1,2,*], Felipe Andres Vinocour Pacheco[2], Jesús Sanchez-Diaz[3], Łukasz Przypis[1,2], Mario Alejandro Mejia Escobar[4], Samy Almosni[2,4], Giovanni Vescio[5], Juan P. Martínez Pastor[6], Blas Garrido[5], Robert Kudrawiec[1], Iván Mora-Seró[3,*] and Senol Öz[4,7,*]

[1]Department of Semiconductor Materials Engineering, Wroclaw University of Science and Technology, Wybrzeze Wyspianskiego 27, 50-370 Wroclaw, Poland

[2]Saule Research Institute, Dunska 11, 54–427 Wroclaw, Poland

[3]Institute of Advanced Materials, Universitat Jaume I, Avenida de Vicent Sos Baynat, 12071 Castelló de la Plana, Spain

[4]Saule Technologies, Dunska 11, 54–427 Wroclaw, Poland

[5]MIND-IN2UB, Department of Electronics and Biomedical Engineering, Universitat de Barcelona, Martí i Franquès 1, 08028 Barcelona, Spain

[6]UMDO, Instituto de Ciencia de los Materiales, Universidad de Valencia, Valencia 46980, Spain

[7]Solaveni GmbH, Siemensstraße 42, 59199 Bönen, Germany

[*]Corresponding authors: wiktor.zuraw@pwr.edu.pl, sero@uji.es, senol.oez@sauletech.com

Materials

Unless otherwise stated, all the materials were purchased from Sigma-Aldrich and used as received. Indium tin oxide (ITO)-coated polyethylene terephthalate (PET) substrates were bought from Eastman Chemical Company. PEDOT:PSS Al 4083 aqueous dispersion and PEDOT:complex HTL Solar 3 dispersion in toluene were purchased from Ossila. Tin(II) iodine (SnI_2, >99.99%) ink was received from Solaveni. Silver (Ag, 99.99%) was purchased from Kurt. J. Lesker. Formamidinium iodide (FAI) was purchased from Ajay North America. Phenethylammonium iodide (PEAI), and butylammonium iodide (BAI) were purchased from Greatcell Solar Materials.

Butylammonium acetate (BAAc) synthesis

Acetic acid and butylamine were mixed in a molar ratio of 1:1. First, butylamine was put into stirring in a round bottom flask in an ice bath, and then a diluted solution of acetic acid in ethanol (1:1, v/v) was dropwise added to the flask under vigorous stirring. After the addition of acetic acid, the reaction was left in the ice bath and continuous steering for 2 hours. After that, the mixture was rotary evaporated at 60 °C for 1 h. The resultant liquid product was put into a fridge for 2 hours to crystallize, and then washed with diethyl ether and recrystallized three to five times. The resultant product was dissolved in absolute ethanol and was rotary evaporated at 60 °C for 1 h. Finally, it was cooled down to room temperature to obtain the final liquid product of BAAc.

Nickel oxide dispersion preparation

Nickel oxide nanoparticles were prepared from a 5 mol L^{-1} aqueous solution of nickel(II) nitrate hexahydrate, tetramethylammonium hydroxide was added to achieve a pH of 10; the precipitated $Ni(OH)_2$ was washed with deionized water five times, then dried at 80 °C and calcinated at 270 °C for 2 h, after which a black fine powder was collected. The suspension was prepared with nanoparticles concentration of 20 mg mL^{-1} in a H_2O:EtOH (85:15 v/v%) mixture.

$(BA_{0.5}PEA_{0.5})_2FA_3Sn_4I_{13}$ precursor solution formulation

First, 3% molar concentration of sodium borohydride $NaBH_4$, 10% molar concentration of SnF_2, and 60 mg mL^{-1} BAAc were added to a 0.6 mol L^{-1} ink of SnI_2 precursor in DMF:DMSO (4:1). Then FAI, BAI, PEAI were added in a (3:1:1) molar ratio and stirred at room temperature until a clear yellow solution was obtained. Before use the solutions were passed through a 0.22 μm PTFE filter.

Module fabrication

ITO-coated PET sheets were patterned using a near-infrared laser (1064 nm wavelength), followed by a washing process with isopropanol. Then, the sheets were dried with N_2 flow and subsequently introduced in an oxygen-plasma chamber for 3 min. The PEDOT:PSS dispersion diluted in ethanol (6:1, v/v), nickel oxide dispersion, or PEDOT:complex dispersion was filtered with a 0.45 μm filter. Then, every dispersion were deposited through a blade-coating technique using an effective gap of 125 μm at a velocity of 2.5 mm·s^{-1} for PEDOT:PSS and nickel oxide dispersions and at a velocity of 5 mm·s^{-1} for the PEDOT:complex dispersion. The obtained films were annealed at 100 °C for 30 min. Additionally, for PEDOT:complex layer a Al_2O_3 wetting layer was deposited using 1 wt% Al_2O_3 dispersion in isopropanol, 250 μm effective gap and 5 mm·s^{-1} velocity. The perovskite deposition was done in N_2 filled glovebox by blade-coating

with an effective gap of 150 μm and a velocity of 0.7 mm·s^{-1}. During the coating, the film was dried with a nitrogen flow through an air knife. Then, the sheets were annealed at 80 ºC for 10 minutes and at 100 ºC for 20 min. A 30 nm layer of C_{60} was thermally evaporated on top of the perovskite layer as the electron transport layer, followed by 5 nm of BCP as a buffer layer. All layers except ITO were patterned (P2) in an ambient atmosphere (~30 min exposure) for the interconnection between cells. Then, 100 nm of Ag was deposited through a shadow mask to obtain the electrical insulation between electrodes of adjacent cells.

Film/module characterization

Scanning electron microscopy

Top-view morphology images of samples were obtained with a field emission SEM (Phenom ProX), which had an accelerating voltage of 10 kV.

X-ray diffraction measurements

X-ray diffraction (XRD) patterns were collected with Rigaku MiniFlex600 (Cu K_α radiation, λ=1.5406 Å) diffractometer.

Current-voltage measurements

J-V measurements were carried out by a Keithley 2461 source measure unit. The solar cells were illuminated with a simulated AM1.5G irradiation of 100 mW/cm^2 using an AAA-rated solar simulator obtained from Abet Technologies, sun 2000, which was calibrated against an RR-208-KG5 silicon reference cell also procured from Abet Technologies. Measurements in low-light conditions were performed under a warm white LED (CLU028-1201C4-303H7M5-F1, Citizen Electronics, ~3280 K). The spectrum of 1000 and 2000 lx illuminance was measured with the spectrometer SEKONIC C-800 Spectromaster and then, proper irradiance was calculated.

Absorption and photoluminescence measurements

Absorption spectra, and steady-state photoluminescence measurements were performed with the Edinburgh Instruments FS5 Spectrofluorometer. Samples were excited with a 405 nm laser diode.

Table S1. P2 processing parameters.

Current	18.8 – 19.8 A
Frequency	3000 Hz
Speed	600 mm/s
Line width	80 µm
Pulse width	1.6 µs

Table S2. Laser power versus output power dependence.

Laser current (A)	18.8	18.9	19.0	19.1	19.2	19.3	19.4	19.5	19.6	19.7	19.8
Output power (W)	0.796	0.835	0.875	0.923	0.971	1.019	1.067	1.115	1.164	1.212	1.260

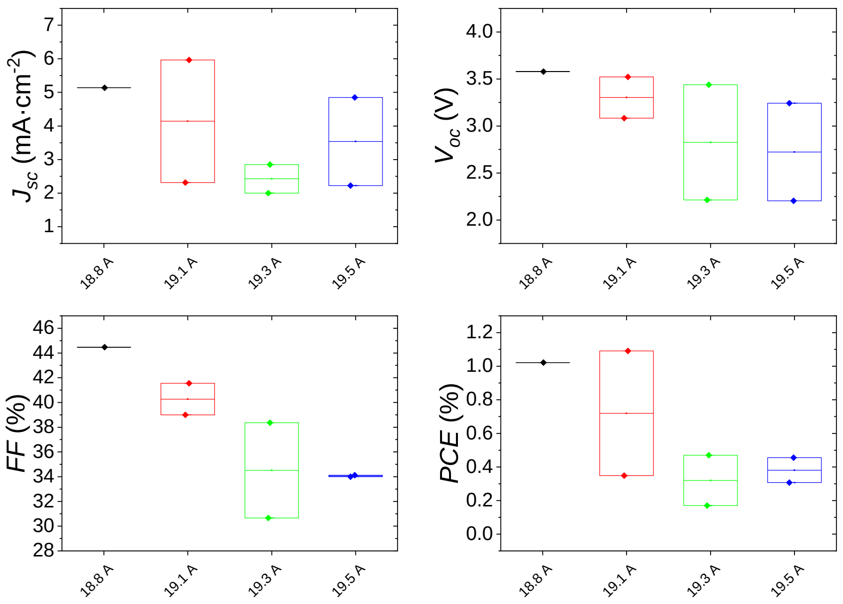

Figure S1. J-V parameters of solar modules fabricated on PEDOT:PSS for different laser operating currents.

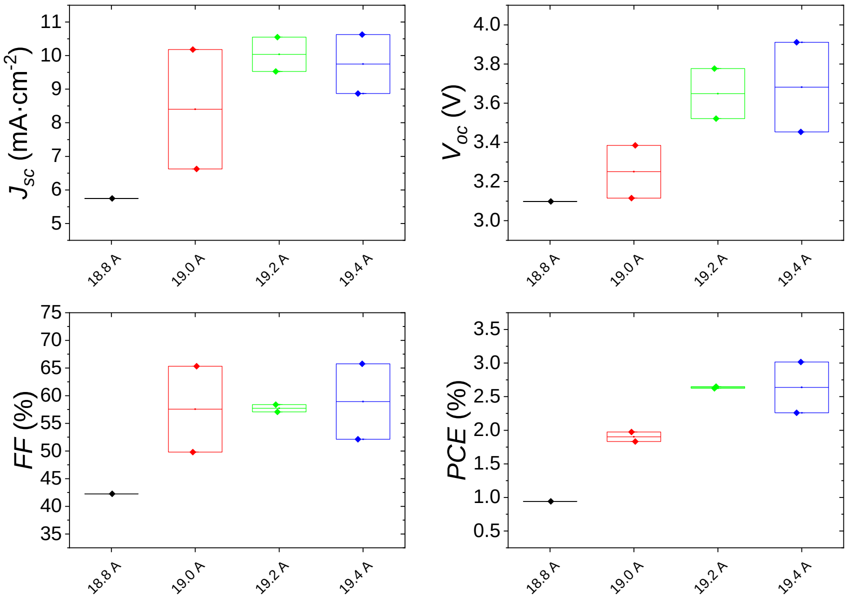

Figure S2. J-V parameters of solar modules fabricated on NiO_x for different laser operating currents.

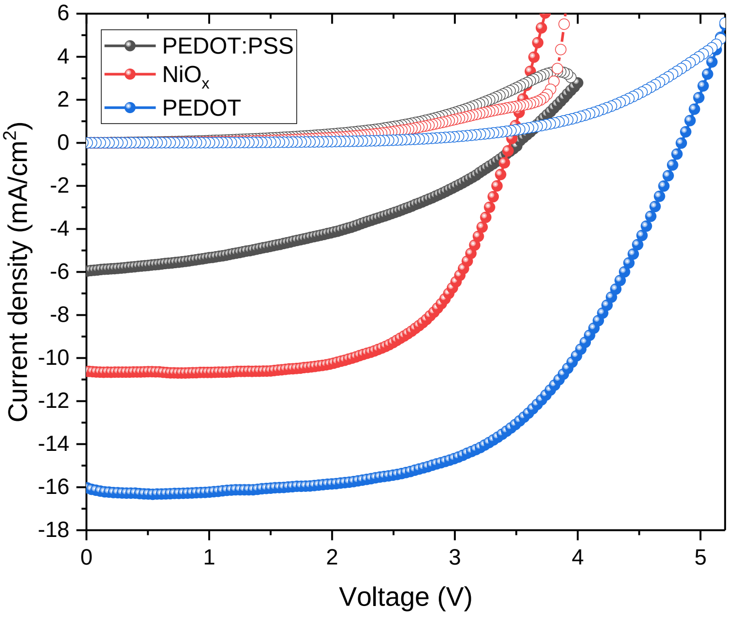

Figure S3. J-V forward scans for different HTMs (filled symbols) and dark scans (unfilled symbols).

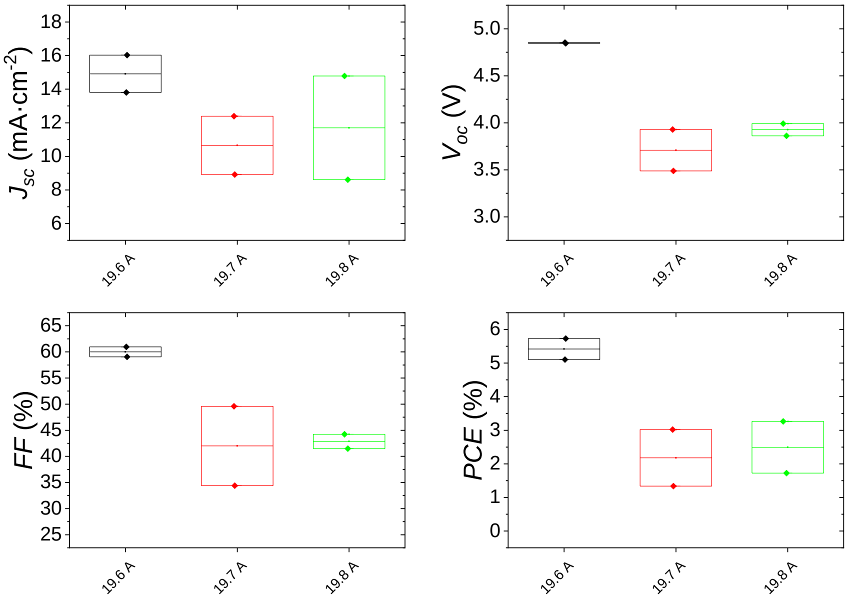

Figure S4. J-V parameters of solar modules fabricated on PEDOT/Al_2O_3 for different laser operating currents.

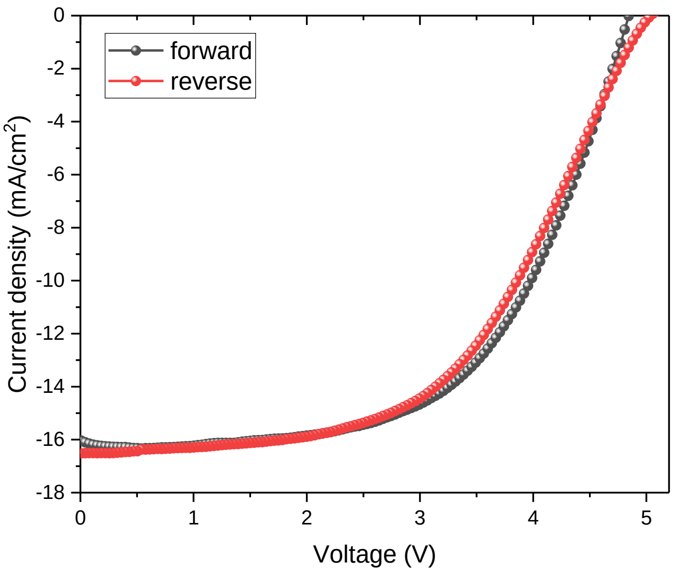

Figure S5. Forward and reverse J-V scan for the champion module.

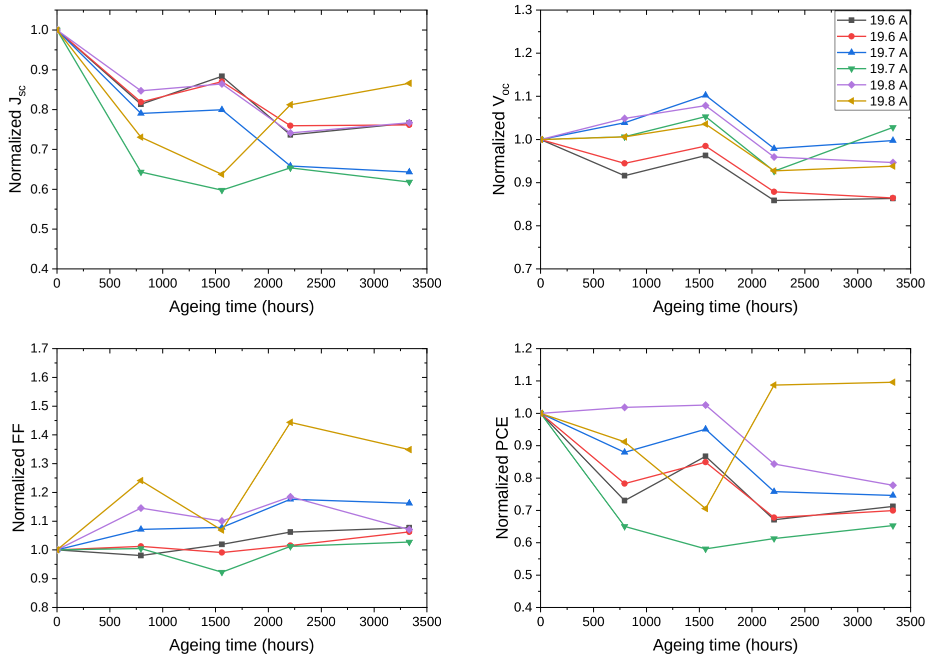

Figure S6. Shelf-stability of modules fabricated on PEDOT/Al_2O_3 for different laser operating currents.

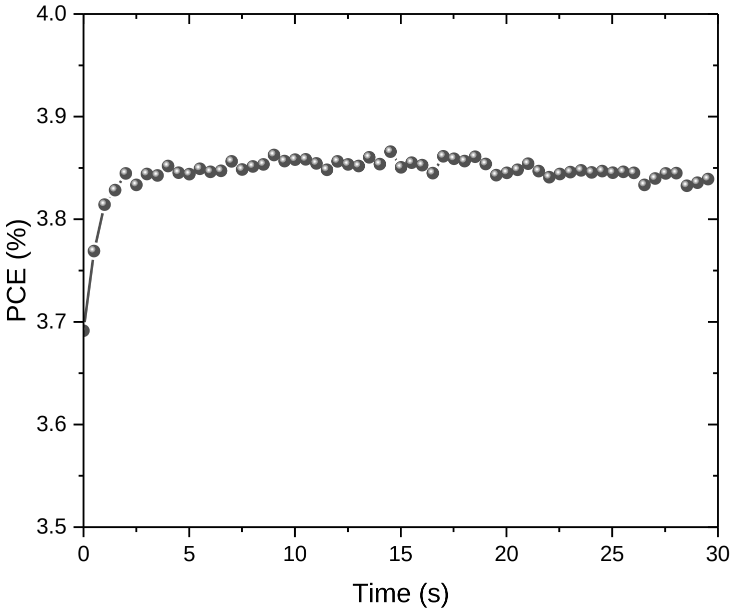

Figure S7. Stabilized power output at maximum power point for the champion module after 3300 hours of storage time (3.94% PCE from J-V scan).

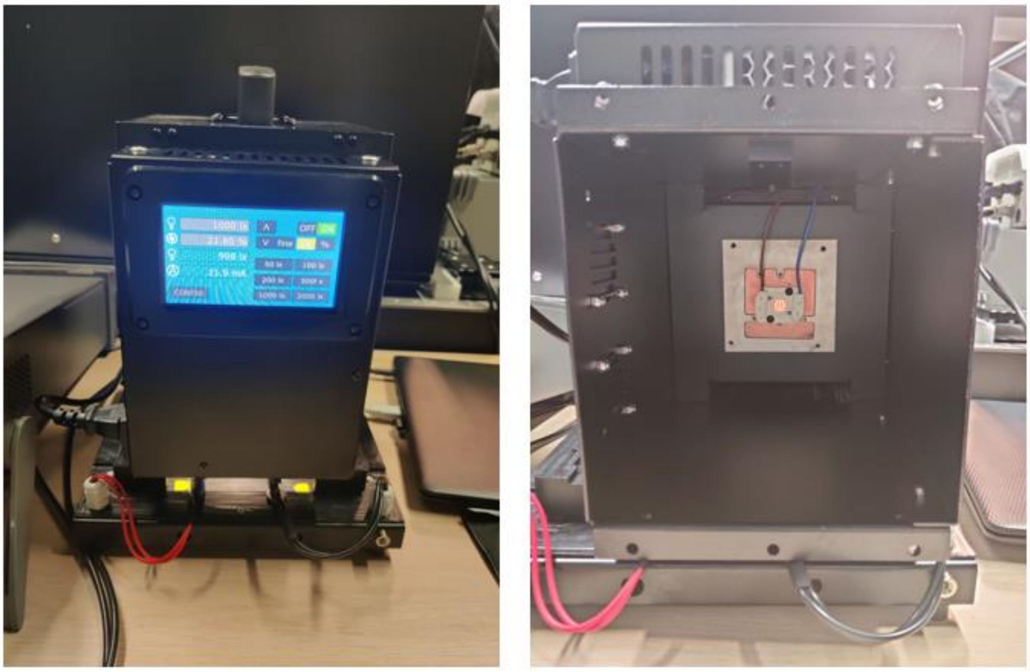

Figure S8. The experimental setup for J-V measurements in low illuminance conditions.

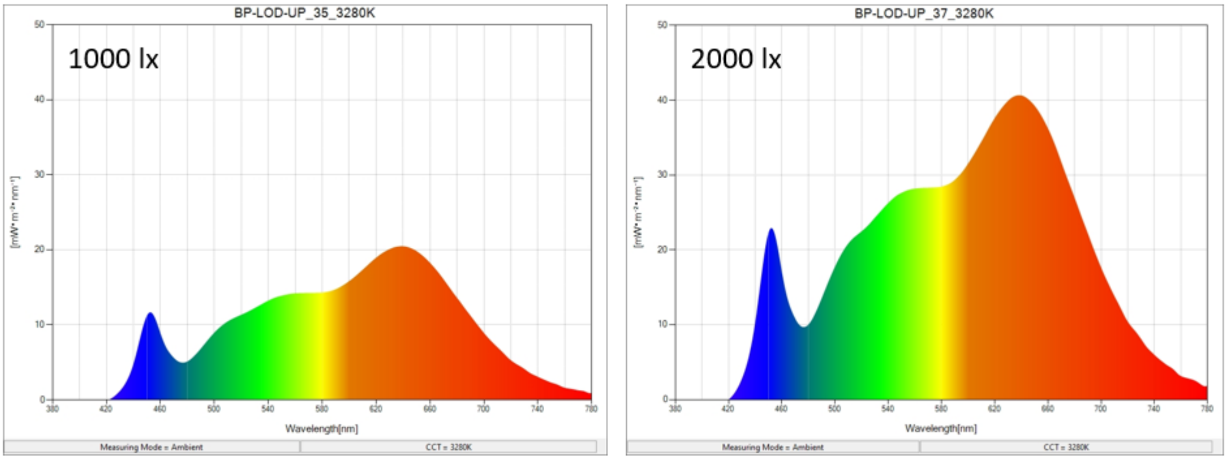

Figure S9. Spectrum of the warm white LED (~3280 K) at 1000 and 2000 lx.
